# Tenofovir Use and Renal Insufficiency among Pregnant and General Adult Population of HIV-infected, ART-Naïve Individuals in Lilongwe, Malawi

**DOI:** 10.1371/journal.pone.0041011

**Published:** 2012-07-27

**Authors:** Derek C. Johnson, Charles Chasela, Madalitso Maliwichi, Albert Mwafongo, Adesola Akinkuotu, Agness Moses, Denise J. Jamieson, Athena P. Kourtis, Caroline C. King, Charlie van der Horst, Mina C. Hosseinipour

**Affiliations:** 1 University of North Carolina Project Malawi, Lilongwe, Malawi; 2 United States Centers for Disease Control and Prevention, Atlanta, Georgia, United States of America; Boston University, United States of America

## Abstract

**Background:**

The Malawian government recently changed its prevention of mother-to-child transmission (PMTCT) regimen and plans to change its first-line antiretroviral therapy (ART) regimen to Tenofovir(TDF)/Lamivudine/Efavirenz as a fixed-dose combination tablet. Implementation could be challenging if baseline creatinine clearance (CrCl) screening were required to assess renal function prior to TDF therapy. Our goal is to determine predictors of CrCl<50 ml/min among HIV-infected, ART-naïve individuals.

**Methodology:**

Data on HIV-infected, ART-naïve adults screened for enrollment into 5 HIV clinical trials in Lilongwe, Malawi were combined for a pooled analysis of predictors for CrCl<50 ml/min. CrCl was derived from the Cockroft-Gault equation. Multivariable logistic regression modeled the association of age, body mass index (BMI), hemoglobin, CD4 cell count <350 cells/mm^3^, gender, and pregnancy with CrCl<50 ml/min.

**Results:**

The analysis included 3508 patients with values for creatinine clearance. Most subjects were female (90.6%) with a median age of 26 years (IQR 22–29). The median CD4 cell count was 444 (IQR 298.0–561.0), and 85.2% percent of women in our study were pregnant. Few patients had CrCl<50 ml/min (n = 38, 1.1%). A BMI less than 18.5 in non-pregnant females (OR = 8.87, 95% CI = 2.45–32.09)) was associated with CrCl<50 ml/min. Hemoglobin level higher than 10 g/dL in males (OR = 0.69, 95% CI = 0.56–0.86) and non-pregnant females (OR = 0.21, 95% CI = 0.04–0.97) was protective against CrCl<50 ml/min.

**Discussion:**

Our findings indicate few patients would be excluded from a TDF-based antiretroviral regimen, suggesting baseline creatinine clearance assessment may not be necessary for implementation. However, in ART settings individuals with low BMI or anemia could potentially be at increased risk for lower CrCl.

## Introduction

Malawi has one of the highest HIV prevalence rates in the world. National HIV adult prevalence in Malawi reached a high of 26% in 1998 and has since decreased to about 12% in 2010 [1]. According to the 2010 United Nations General Assembly Special Session (UNGASS) *Country Progress Report*, over 930,000 Malawians currently live with HIV and approximately 84,000 new HIV infections occur annually [2]. Since 2004, the Malawian Ministry of Health has provided free antiretroviral therapy (ART) to individuals living with HIV and eligible for treatment through the Global Fund to Fight AIDS [3].

Malawi's current first-line antiretroviral therapy and former prevention of mother-to-child transmission (PMTCT) regimen consists of stavudine, lamivudine and nevirapine. Stavudine has long-term side-effects such as lactic acidosis, hepatic steatosis and lipo-atrophy [4]. As such, the Malawian government plans to change the first-line ART regimen for all individuals living with HIV to tenofovir disoproxil fumarate (TDF)/lamivudine/Efavirenz as a fixed-dose, combination tablet in accordance with WHO guidelines, despite the uncertainty of possible negative health effects associated with efavirenz use during pregnancy [5]. TDF has an excellent safety profile with rare reports of nephrotoxicity, proteinuria, and renal tubular dysfunction with Fanconi syndrome [6], [7], and its safety as a first-line ARV medication has been demonstrated in other African countries [8].

A creatinine clearance (CrCl) rate ≥50 ml/min is recommended before starting TDF for efficient renal clearance [9], [10]. In Malawi, previous studies among individuals living with HIV suggest that approximately 18.8% have a CrCl of 30–59 ml/min and 2.2% have a CrCl less than 30 ml/min [11]. While recommendations exist for dose modification in patients with lower creatinine clearance [12], these modifications preclude the use of a convenient fixed-dose, combination tablet. Given the limited laboratory infrastructure in Malawi, performing routine baseline CrCl screening could hinder the implementation of TDF as a component of first-line ART. As such, the goal of the current analysis is to determine prevalence and predictors of CrCl<50 ml/min among HIV-infected, ART-naïve individuals in Lilongwe, Malawi.

## Methods

### Study Design

All involved studies were approved by the National Health Sciences Research Committee, the University of North Carolina Ethics Committee. The BAN study was also approved by CDC ethics committee. All subjects provided written informed consent for the participation in the specified clinical trial that included data collected from screening onward.

This cross-sectional study combines data on 3508 HIV-infected, ART-naïve adults screened for enrollment to five HIV clinical trials conducted in Lilongwe, Malawi from 04/08/2004 to 07/26/2009: 625 screened for the HIV Prevention Trial Network 052 (HPTN 052: ClinicalTrials.gov Identifier NCT0007458); 268 for the AIDS Clinical Trial Group (ACTG) A5175 (ClinicalTrials.gov Identifier NCT00096824), A5208 (ClinicalTrials.gov Identifier NCT00089505), A5221 (ClinicalTrials.gov Identifier NCT00108862); and 2615 for the Breastfeeding, Antiretroviral, and Nutrition Study (BAN: ClinicalTrials.gov Identifier NCT00164736) [13], [14]. All study participants had non-missing values for serum creatinine, age, weight, and gender; the necessary components to calculate a Cockroft-Gault creatinine clearance. Screened individuals were drawn from the general HIV infected population. There were no exclusion criteria that would have excluded patients prior to our evaluation based on co-morbid diseases associated with kidney disease. Data was collected at screening before an individual was selected for recruitment into a clinical trial. Using screening data allowed us to capture a broad range of individuals and reduced selection bias based on pre-existing co-morbidities.

### Independent Variables and Covariate Definitions

The outcome for this analysis was CrCl<50 ml/min based on dichotomization of CrCl calculated from the Cockroft-Gault equation (CG): Creatinine clearance = [(140-age)*(weight in Kg)*(0.85 if female)]/(72*Creatinine mg/dL) [15]. All trials included in this analysis collected information at screening on serum creatinine, CD4 cell count, and hemoglobin. Measurements of serum creatinine as a part of routine care are not available in the public sector of care in Malawi, nor through the clinics that referred clients to us. Hemoglobin was categorized as being greater than or equal to versus less than 10 g/dL for both descriptive purposes and in our models. Body mass index (BMI) was categorized as greater than or equal to 18.5 versus less than 18.5 which is the standard endpoint for an underweight classification according to Malawi guidelines. CD4 cell count was categorized as greater than compared to less than or equal to 350 cells/mm^3^, the threshold for antiretroviral (ART) initiation according to Malawi guidelines. The eligibility criteria for initiating ART in Malawi is a CD4 cell count of less than 350 cells/mm^3^ or a WHO stage 3 or 4 defining condition. The PMTCT regimen for Malawi states that all pregnant women regardless of status are eligible for ART. Given there is a 40–50% increase in serum creatinine in pregnant women [16], [17], a sensitivity analysis using CrCl<90 ml/min in pregnant women as an outcome was conducted.

### Statistical Analysis

Bivariate analyses were used to assess the crude association for each covariate with CrCl<50 ml/min according to both CrCl equations. Differences in categorical variables were assessed using the χ^2^-square test, while differences in means were assessed using the student's t test and Wilcoxon rank-sum test. Descriptive characteristics were stratified by pregnancy status due to the large amount of pregnant women in our study. Analysis of variance was used to assess the homogeneity of each covariate across each clinical trial. Multivariable logistic regression using SAS 9.2 assessed risk factors associated with CrCl<50 ml/min, while adjusting or stratifying on screening population. A sensitivity analysis assessing CrCl of <90 ml/min in pregnant women was conducted to assess the robustness of CrCl in pregnant women in our models.

## Results

Most study subjects were female (90.6%) with a median age of 26 years (IQR 22–29). The median CD4 cell count was 444 cells/mm^3^ (IQR 298.0–561.0). The median weight of our study participants (57 kg (IQR 52–63)) is comparable to a study of 4 ART clinics where the median weight of individuals seeking ARVs was 55 kg (IQR 49–63) [18]. The majority of our study population was pregnant (85.2%), due to all of the participants from the BAN clinical trial being pregnant as well as some ACTG and HPTN 052 clients. Descriptive statistics of the BAN clinical trial also describe the characteristics of pregnant individuals. Tests of equality among baseline demographics across each of the three separate clinical trial datasets indicated that covariates differed significantly across each trial (Table 1).

**Table 1:** Baseline demographic and clinical characteristics of HIV-infected, ART-naïve individuals at screening visit, stratified by clinical trial and pregnancy status.

	BAN n = 2615		Combined ACTG n = 268		HPTN052 n = 625	
	Not Pregnant (N = N/A)	Pregnant (N = 2615)	Not Pregnant (N = 204)	Pregnant (N = 14)	Not Pregnant (N = 550)	Pregnant (N = 75)
Age						
Mean (SD)	N/A	24.7 (4.5)	22.9 (13.4)	31.2 (5.7)	34.4 (9.9)	26.7 (5.4)
Median (IQR)	N/A	24.0 (21.1–27.0)	20.0 (15.0–26.0)	30.0 (28.0–34.0)	33.0 (27.0–40.0)	26.0 (22.0–30.0)
Gender						
Female	N/A	2615 (100.0)	188 (74.0)	14 (100.0)	280 (50.9)	75 (100.0)
Male	N/A	N/A	66 (26.0)	N/A	270 (49.1)	N/A
BMI (kg/m^2^)						
Mean (SD)	N/A	24.1 (2.9)	21.3 (3.4)	21.5 (3.6)	21.9 (3.3)	23.6 (3.1)
Median (IQR)	N/A	23.7 (22.2–25.6)	20.7 (19.1–22.0)	20.9 (19.4–22.6)	21.2 (19.6–23.5)	22.9 (21.0–24.9)
BMI (kg/m^2^)						
<18.5	N/A	844 (32.3)	49 (19.3)	2 (14.3)	75 (13.6)	5 (6.7)
≥18.5	N/A	1771 (67.7)	205 (80.7)	12 (85.7)	475 (86.4)	70 (93.3)
Hemoglobin (g/dL)						
<10	N/A	622 (23.8)	45 (17.8)	2 (14.3)	43 (7.9)	13 (17.3)
≥10	N/A	1993 (76.2)	208 (82.2)	12 (85.7)	500 (92.1)	62 (82.7)
Hemoglobin (g/dL)						
Mean (SD)	N/A	10.8 (1.2)	11.4 (2.2)	12.3 (1.6)	12.8 (2.1)	11.2 (1.9)
Median (IQR)	N/A	10.8 (10.0–11.7)	11.6 (10.4–12.9)	12.6 (11.2–13.5)	12.8 (11.4–14.3)	11.4 (10.3–12.2)
CD4+ T-cell count (cells/mm^3^)						
Mean (SD)	N/A	482.7 (198.2)	255.6 (195.5)	330.6 (175.2)	365.2 (233.9)	372.5 (183.6)
Median (IQR)	N/A	441.0 (334.0–585.0)	212.5 (121.0–339.0)	288.5 (237.0–422.0)	324.5 (206.0–502.0)	355.5 (219.0–490.0)
CD4+ T-cell count (cells/mm^3^)						
>350	N/A	1838 (70.3)	196 (77.2)	8 (57.1)	297 (54.0)	36 (48.0)
≤350	N/A	777 (29.7)	58 (22.8)	6 (42.9)	253 (46.0)	39 (52.0)
ALT (U/L)						
Mean (SD)	N/A	14.1 (5.8)	22.9 (13.4)	244.1 (68.2)	23.7 (13.4)	14.6 (5.0)
Median (IQR)	N/A	13.0 (11.0–16.0)	20.0 (15.0–26.0)	235.0 (201.0–289.0)	20.0 (15.0–27.0)	14.0 (12.0–17.0)
Creatinine Clearance <50 ml/min						
Yes	N/A	9 (0.3)	9 (3.5)	1 (7.1)	19 (3.5)	0 (0.0)
No	N/A	2606 (99.7)	245 (96.5)	13 (92.9)	531 (96.5)	75 (100.0)
Creatinine Clearance in Pregnant Females <90 ml/min						
Yes	N/A	410 (15.7)	N/A	11 (78.6)	N/A	8 (10.7)
No	N/A	2205 (84.3)	N/A	8 (21.4)	N/A	67 (89.3)

Few patients had CrCl<50 ml/min by the CG equation (n = 38, 1.1%). The proportion with CrCl<50 ml/min differed by study population (Table 1). Among a subgroup of patients with a CD4 cell count less than 350 (n = 1314), approximately 1.9% experienced CrCl<50 ml/min. Among individuals with a CD4 cell count less than 200 (n = 289), approximately 6.9% have CrCl<50 ml/min. Each one-year increment in age was associated with increased odds of CrCl<50 ml/min in the BAN and HPTN 052 clinical trials in both univariate (Table 2) and multivariable (Table 3) analysis. When stratified by gender and pregnancy (Table 3), each one-year increment in age was associated with increased odds for CrCl<50 ml/min, men (OR = 1.08, 95% CI = 1.02–1.14), non-pregnant females (OR = 1.17, 95% CI = 1.08–1.26), and pregnant females (OR = 1.15, 95% CI = 1.04–1.27) (Table 4).

**Table 2:** Crude and Adjusted odds ratios (ORs) for potential predictors of creatinine clearance <50 mg/dl among HIV-infected, ART-naïve individuals, stratified by clinical trial.

	BAN		Combined ACTG		HPTN052	
	Crude OR (95% CI)	Adjusted OR (95% CI)	Crude OR (95% CI)	Adjusted OR (95% CI)	Crude OR (95% CI)	Adjusted OR (95% CI)
Age (per year increase)	1.15 (1.03–1.27)	1.14 (1.03, 1.28)	1.07 (0.98–1.16)	1.07 (0.98, 1.16)	1.10 (1.06–1.15)	1.13 (1.07, 1.19)
Gender						
Female	N/A	N/A	1.32 (0.33–5.28)	1.75 (0.34, 9.07)	2.94 (1.10–7.84)	1.19 (0.35, 4.07)
Male	N/A	N/A	Ref	Ref	Ref	Ref
BMI (kg/m^2^)						
<18.5	1.68 (0.45–6.28)	1.68 (0.44, 6.30)	4.61 (1.28–16.58)	4.27 (1.07, 17.11)	5.40 (2.10–13.86)	5.23 (1.77, 15.45)
≥18.5	Ref	Ref	Ref	Ref	Ref	Ref
Hemoglobin (per g/dL increase)						
≥10	1.09 (0.23, 5.27)	1.10 (0.22, 5.43)	0.30 (0.8, 1.11)	0.34 (0.08, 1.38)	0.12 (0.5, 0.31)	0.07 (0.02, 0.23)
<10	Ref	Ref	Ref	Ref	Ref	Ref
CD4+ T-cell count (cells/mm^3^)						
>350	1.48 (0.31–7.14)	1.53 (0.31, 7.51)	0.34 (0.04–2.77)	0.44 (0.05, 3.74)	0.52 (0.19–1.38)	0.84 (0.27, 2.67)
≤350	Ref	Ref	Ref	Ref	Ref	Ref
Currently Pregnant*						
No	N/A	N/A	0.48 (0.06, 4.06)	0.42 (0.04, 4.58)	N/A	N/A
Yes	N/A	N/A	Ref	N/A	N/A	N/A

Models adjusted for age, gender, BMI, hemoglobin, CD4, and pregnancy.

* No pregnant women in the HTPN 052 clinical trial experienced creatinine clearance <50 mg/dl. All women screened for the BAN clinical trial were pregnant.

**Table 3:** Adjusted odds ratios (aORs) for potential predictors of Creatinine Clearance <50 mg/dl in HIV-infected, ART-naïve individuals, stratified by gender and pregnancy.

	Male (N = 336)		Non-Pregnant Females (N = 468)		Pregnant Females (N = 2704)	
	Crude OR (95% CI)	Adjusted OR (95% CI)	Crude OR (95% CI)	Adjusted OR (95% CI)	Crude OR (95% CI)	Adjusted OR (95% CI)
Age (per year increase)	1.07 (1.02, 1.12)	1.08 (1.02, 1.14)	1.12 (1.06, 1.18)	1.17 (1.08, 1.26)	1.14 (1.04, 1.27)	1.15 (1.04, 1.27)
BMI (Kg/m^2^)						
<18.5	2.68 (0.94, 7.67)	1.67 (0.52, 5.38)	7.63 (2.39, 24.67)	8.87 (2.45, 32.09)	2.18 (0.63, 7.57)	2.26 (0.65, 7.86)
≥18.5	Ref	Ref	Ref	Ref	Ref	Ref
CD4+ T-cell count						
>350 cells/mm^3^	0.49 (0.13, 1.77)	0.78 (0.19, 3.20)	0.62 (0.18, 2.08)	0.73 (0.16, 3.44)	1.02 (0.26, 3.94)	1.04 (0.26, 4.11)
≤350 cells/mm^3^	Ref	Ref	Ref	Ref	Ref	Ref
Hemoglobin (per g/dL increase)						
≥10	0.10 (0.04, 0.29)	0.69 (0.56, 0.86)	0.23 (0.07, 0.80)	0.21 (0.04, 0.97)	1.23 (0.26, 5.82)	1.34 (0.28, 6.48)
<10	Ref	Ref	Ref	Ref	Ref	Ref

Models adjusted for Age, BMI, CD4 cell count, and hemoglobin.

**Table 4:** Crude and adjusted odds ratios (ORs) for sensitivity analysis of potential predictors of creatinine clearance <90 ml/min among HIV-infected, ART-naïve pregnant women, stratified by clinical trial.

	BAN (N = 2615)*		Combined ACTG (N = 14)		HTPN 052 (N = 75)	
	Crude OR (95% CI)	Adjusted OR (95% CI)	Crude OR (95% CI)	Adjusted OR (95% CI)	Crude OR (95% CI)	Adjusted OR (95% CI)
Age (per year increase)	1.12 (1.09, 1.14)	1.12 (1.09, 1.14)	1.78 (0.91, 3.50)	1.07 (1.02, 1.13)	1.10 (0.96, 1.26)	1.10 (1.08, 1.12)
BMI (kg/m^2^)						
<18.5	1.19 (0.95, 1.49)	1.20 (0.96, 1.51)	1.20 (0.85, 1.72)	1.31 (1.18, 1.45)	2.25 (0.22, 23.0)	6.70 (3.34, 13.41)
≥18.5	Ref	Ref	Ref	Ref	Ref	Ref
Hemoglobin (per g/dL increase)						
≥10	0.63 (0.49, 0.79)	0.65 (0.51, 0.83)	0.71 (0.28, 1.81)	0.67 (0.30, 1.49)	1.02 (0.70, 1.50)	1.44 (0.76, 2.69)
<10	Ref	Ref	Ref	Ref	Ref	Ref
CD4+ T-cell count (cells/mm^3^)						
>350	1.38 (1.11, 1.72)	1.32 (1.06, 1.67)	1.67 (0.12, 24.25)	1.19 (0.60, 2.39)	3.09 (0.58, 16.42)	1.01 (0.71, 1.45)
≤350	Ref	Ref	Ref	Ref	Ref	Ref

Models adjusted for age, BMI, hemoglobin, CD4 cell count.

* All women screened for the BAN clinical trial were pregnant.

CrCl<50 ml/min was associated with BMI and hemoglobin level. A BMI ≤18.5 kg/m^2^ was significantly associated with increased odds for CrCl<50 ml/min in both the HPTN 052 and combined ACTG trials in univariate analysis (HPTN 052: OR = 5.40, 95% CI = 2.10–13.86, p-value≤0.001; ACTG: OR = 4.61, 95% CI = 1.28–16.58, p-value≤0.05; Table 2) and in the in multivariable analysis (HPTN 052: OR = 5.23, 95% CI = 1.77–15.45; ACTG: OR = 4.27, 95% CI = 1.07–17.11; Table 2). When stratified by gender and pregnancy, BMI ≤18.5 kg/m^2^ was significantly associated with increased odds of CrCl<50 ml/min in non-pregnant females (OR = 8.87, 95% CI = 2.45–32.09; Table 3). Hemoglobin greater than 10 g/dL was protective against CrCl<50 ml/min for both men (OR = 0.69, 95% CI = 0.56–0.86; Table 3) and non-pregnant women (OR = 0.21, 95% CI = 0.04–0.97; Table 3).

Sensitivity analyses assessed factors associated with CrCl<90 ml/min in pregnant individuals by clinical trial while adjusting for age, BMI, hemoglobin, and CD4 cell count was similar to our main models. Covariates in our sensitivity analysis that differed from our main models varied according to clinical trial. Being underweight was associated with CrCl<90 ml/min in the combined ACTG trials (OR = 1.31 95%CI 1.18–1.45), but not associated with CrCl<50 ml/min in the combined ACTG trials of our main models. Hemoglobin values greater than 10 g/dL was protective against a CrCl<90 ml/min in the BAN trials (OR = 0.65 95%CI = 0.51–0.83), but only significantly associated with CrCl<50 ml/min in the HPTN052 trial of our main models. CD4 cell count was found to be protective against CrCl<90 ml/min in the BAN trials (OR = 1.31 95%CI 1.18–1.45), but not associated with CrCl<50 ml/min in any trial of our main models (Table 4).

## Discussion

Among 3508 HIV-infected, ART-naïve adults screened for enrollment to five HIV clinical trials conducted in Lilongwe, Malawi, we found that CrCl<50 ml/min was rare, suggesting that few patients would be excluded from receiving the public provision of TDF-based antiretroviral regimen based on this criteria. The small proportion of patients with low CrCl is comparable to other studies assessing CrCl in HIV positive individuals in populations similar to ours [19] Our pooled study population provides the most comprehensive and representative data on CrCl among HIV-infected, ART-naïve patients eligible for PMTCT or ART in Lilongwe, since screening recruitment for each trial had an extensive outreach component targeting PMTCT or ART eligible individuals. The pooled data indicate that most eligible HIV-infected, ART-naïve patients would not be excluded from the proposed TDF-based, first-line ART and PMTCT regimen based on CrCl criteria. Therefore, broad implementation of a fixed-dose, combination tablet as a first-line ART likely does not require assessment of baseline CrCl in resource-constrained settings, particularly for healthy pregnant females.

Many indicators suggest our population is comparable to other HIV-infected, ART-naïve cohorts within Malawi. Studies conducted in the PMTCT populations of Thyolo District Hospital located in rural Malawi have shown the median age of PMTCT study participants is 22 years, making our study sample of comparable age to other Malawian PMTCT cohorts [20]. A large study of over 13,000 individuals at two ART clinics in Lilongwe indicated that the majority of individuals seeking care were young females. While our study oversamples females due to using data from the BAN clinical trial, females represent 60% of those seeking ART nationally and higher proportions are expected when PMTCT is used as an entry point to ART.

Our cohort's median CD4 cell count, at 444 cells/ml, is higher than most studies assessing risk factors associated with low creatinine clearance in HIV-infected individuals [21], [22] but is typical of CD4 counts among pregnant women attending ANC clinics [23]. While a higher CD4 cell count suggests a healthier population, we found a low rate of CrCl<50 ml/min even among those with CD4 counts below 350 cells/mm^3^ (1.9%), and below 200 cells/mm^3^ (6.9%).

Our study's association between low BMI and CrCl<50 ml/min agrees with the current literature on renal insufficiency among HIV-infected, ART-naïve individuals. A cross-sectional survey of 2588 individuals assessing creatinine clearance in HIV-infected patients in France found that a BMI<22 kg/m^2^ was associated with both mild and advanced renal insufficiency [24]. Other studies have found significant differences in the BMI of individuals with HIV-associated nephropathy and those without [25]. Our sensitivity analysis showed CrCl<90 ml/min in pregnant women was associated with being under weight. This suggests that lower BMI is associated with a lower CrCl even after considering the increase in serum creatinine in pregnant women.

Low hemoglobin values are associated with renal insufficiency in people living with HIV. Time to death due to end-stage renal disease in individuals living with HIV is significantly shorter for those with low hemoglobin values compared to normal values [26]. Kidney-related morbidities, such as albuminuria and renal hyperfiltration, are also highly correlated with HIV infection and low hemoglobin [27]. Both our main models and our sensitivity analysis for the BAN clinical trial found that lower hemoglobin values were associated with increased odds of renal insufficiency. This is consistent with current literature on hemoglobin and kidney function in HIV-infected individuals [28].

Our findings show increases in hemoglobin and BMI have significant protective affects against CrCl<50 ml/min is consistent with other literature. HIV/AIDS-associated nephropathy (HIVAN) is associated with low hemoglobin levels [28]. Additionally, the effect of BMI on renal impairment has been shown to differ with respect to gender (22).

Models stratified by pregnancy show only increases in age to be a significant factor associated with CrCl<50 mg/dl in ART-naïve pregnant individuals, while lower BMI and hemoglobin are associated with a increased likelihood of CrCl<50 mg/dl for non-pregnant individuals. The lack of significant factors associated with CrCl<50 mg/dl and pregnancy could possibly be due to the renal changes that occur during pregnancy [29], [30], [31]. BMI, hemoglobin, and serum creatinine levels change during pregnancy [32], [33] and these changes could possibly affect creatinine clearance.

While CD4 cell count was not significant in our multivariate models, 20 of the 38 individuals with CrCl<50 ml/min using the CG equation also had CD4 counts less than 200 cells/mm^3^ (52.6%). Of the 38 individuals, 11 had a BMI under 18.5 (28.9%), and 7 of these also had a hemoglobin <11 g/dl and CD4 <200 cells/mm^3^. This suggests that when clinically assessing patients for potential CrCl<50 ml/min in a general clinical setting, a disproportionate number of afflicted individuals (18.4%) will have low hemoglobin, BMI, and CD4 cell counts. While we were not able to develop a clinical algorithm for assessing CrCl<50 ml/min due to small numbers of clients with this outcome, developing and validating such an algorithm prospectively with a larger study sample could be possible.

There are some limitations to our study. We recognize that the CG equation does not addresses renal tubular function as can be assessed by evaluating proteinuria through dipstick, urine protein∶creatinine ratio, or fractional excretion of phosphate. The clinical trials included in this pooled analysis were all conducted in the urban city of Lilongwe and may not be representative of all the PMTCT and ART populations in Malawi. A recent WHO report suggests that only 17% of Malawi's population live in urban areas [34]. However, most HIV infections occur in Malawi's cities [34].

Enrollment criteria for the respective trials varied, and if CD4 clinic data were available and it precluded patients from the study, they would not have been referred for trial screening. The ACTG 5221 and ACTG 5208 trials did not screen individuals with known high CD4 counts, and the BAN study did not enroll individuals with known CD4<250 cells/µL. Therefore, the pooled population may have oversampled sicker individuals for the ACTG screening population and healthier individuals for the BAN screening population. Such misrepresentation is expected to be minimal as individuals presenting for ART tend to be sicker on average and those presenting for PMTCT tend to be healthier. However, it is possible patients screened for the five clinical trials may not be representative of a routine clinic setting because screened individuals might be healthier than normal.

The majority of our population consists of pregnant women screened through the BAN study. This could potentially create an underrepresentation of HIV-infected, ART-naïve men. However, a main goal of our study is to focus on predictive factors of potential renal failure for pregnant mothers in a PMTCT program. Additionally, regardless of the gender imbalance across studies, the data used in this pooled analysis is, to our knowledge, the only data containing serum creatinine values of HIV-infected, ART-naïve individuals in Malawi. It is possible the CG equation could misrepresent creatinine clearance in our study because the majority of our population is pregnant and the equation requires the weight and gender of each subject. To address this potential limitation, we included a sensitivity analysis using CrCl<90 ml/min in pregnant individuals as an outcome.

Our study suggests that few eligible HIV-infected Malawians would be excluded from a TDF-based ART regimen. Limited resources for measuring serum creatinine should be directed towards individuals with low values for BMI or hemoglobin and who are not pregnant, since these individuals are at the greatest risk for a CrCl<50 ml/m.
